# New Trends in Pediatric Hospitalizations for Acute Intoxications: A Single-Center Long-Term Retrospective Study

**DOI:** 10.3390/children12060701

**Published:** 2025-05-29

**Authors:** Ivona Vrkić Boban, Marijan Saraga

**Affiliations:** 1 Department of Pediatrics, University Hospital of Split, Spinčićeva 1, 21000 Split, Croatia; 2 School of Medicine, University of Split, Šoltanska 2, 21000 Split, Croatia; marijan.saraga@gmail.com

**Keywords:** alcohol, intoxications, children, drugs, medicaments

## Abstract

**Background**: Alcohol and drugs are the most common causes of acute intoxications in children. The aim of our study was to determine changes in hospitalizations for acute intoxications, especially acute alcohol intoxications (AAIs), among children aged 0–18 at the Department of Pediatrics, University Hospital of Split from 2016 to 2021 and to compare them to results of the previous studies conducted in the same department. **Methods**: We collected data from hospital medical records from 1 January 2016 to 31 December 2021. According to the cause of intoxication, children were divided into two groups, those with AAIs and those with non-alcohol intoxications (non-AAIs), and four age groups, 0–5, 6–9, 10–13, and 14–18 years. We used SPSS 25 for statistical analyses with a *p* value < 0.05 as statistically significant. **Results**: There were 218 children hospitalized for acute intoxications at the Department of Pediatrics, University Hospital of Split, 71 for AAIs and 147 due to intoxications with other substances. Medicaments were the most common cause of acute intoxications (41.29%), followed by alcohol (32.57%). Among children hospitalized for drugs intoxications, there were 18.89% suicide attempts, and 88.23% of them were girls. Non-AAIs were most common among 0–5- and 14–18-year-olds. AAIs were most common among boys who were 14–18 years old. The average blood alcohol concentration was 2.15‰, and 8.45% children tested positive for drugs. A significant decrease in AAIs among all hospitalizations was detected in the observed period, especially during COVID-19, predicting a further annual decrease of 21.26%. **Conclusions**: Although hospitalizations for AAIs among children are decreasing, increasing intoxications with non-medical drugs and medicaments, especially those intentional as suicidal attempts, indicate that intensive work on the mental health of young people and children is necessary.

## 1. Introduction

Intoxication is a sudden threat of one or more organic systems, caused by contact with a toxic substance [1]. Alcohol and drugs are the most common causes of acute intoxications in children [2,3,4]. Intentional intoxications are more often seen among older children, especially girls, for problems with parents or in school [5,6], while accidental intoxications are most often seen among boys of younger ages [6].

Alcohol absorption starts 10 min after its consummation and reaches the highest blood concentration after 30–90 min [7]. Alcohol stimulates dopamine excretion, causing euphoria [8], but it also has anxiolytic effect [8] and causes temporary anterograde amnesia [7]. The first symptoms of acute drunkenness are euphoria and “good mood” [8]. Severe alcohol intoxication is defined with blood alcohol intoxication (BAC) of 2.50 mg/g [9] because coma usually occurs at BAC of 2.0 to 2.5 mg/g [8]. Alcohol intoxication in children results in consciousness disorders, hypothermia, and hypoglycemia more often than it does in adults [10], also resulting in electrolytes misbalance [11,12], metabolic acidosis [13], and liver damage [14]. Hypoglycemia is most often seen in children younger than 5 [11]. The most severe consequence of alcohol consumption is respiratory depression [7]. Symptoms of acute alcohol intoxication (AAI) in children can occur with lower BAC [9] and more often with symptoms of the central nervous system than in adults because their body contains more water than the body of an adult person [9]. Frequent consumption of alcohol and marijuana during adolescence can lead to brain damage [15,16].

Hospitalizations for AAIs among children have changed over time, with some studies reporting an increasing trend [3,17,18,19,20,21,22,23,24], and others a decreasing one [22,25,26,27]. Similar to those findings, a study provided at the Department of Pediatrics, University Hospital of Split found an alarming increasing trend of AAIs among children over the period from 1997 to 2007 [3]. On the other hand, another study at the same department found a significant deceasing trend from 2008 to 2015, predicting a further annual decline of 10.10% [26]. The aim of our study was to explore if the predicted declining trend really continued from 2016 to 2021, or some changes had happened, with our special attention given to a period of the COVID-19 pandemic in the year 2020.

## 2. Material and Methods

### 2.1. Data Collection

We collected data retrospectively from hospital medical records and medical histories at the Department of Pediatrics, University Hospital of Split over the period from 1 January 2016 to 31 December 2021. During that period, 13,772 children were hospitalized at the Department of Pediatrics, University Hospital of Split, 218 of them for acute intoxication. We collected data of age, gender, and substance that caused intoxication, and for children with alcohol intoxications, we collected BAC, month and day of intoxication (working days or weekends and holidays), location where intoxication had happened (at home or outside), type of consumed alcoholic beverages that led to intoxication (wine/beer, shots, or mixed drinks), presence of injuries, presence of drugs and medications in urine samples, and the interventions of a psychologist or psychiatrist.

### 2.2. Participants

All children with anamnestic or hetero-anamnestic data of ingestion of toxic substances, as well as those with symptoms of intoxication, were received through our receiving department. After administration, clinical examination and blood and urine samples were taken to detect BAC and presence of drugs and medications. According to the cause of intoxications, children were divided into two groups: AAIs (those who became intoxicated with alcohol) and non-AAIs (all other types of intoxications, e. g, drugs, chemicals, non-medical drugs, poisons etc.). They were divided into four age groups, depending on the educational program they were attending, as in previous studies [3,26]: 0–5 years (preschool children), 6–9 years (early elementary school students), 10–13 years (senior elementary school students), and 14–18 years (high school students).

### 2.3. Statistical Analyses

We used SPSS 25 (IBM, Armonk, New York, NY, USA; 2017) for statistical analysis. Numeric variables were shown as mean and standard deviation or median and interquartile range, depending on the distribution, which was previously tested by the Kolmogorov–Smirnov test. Categorical variables were shown as absolute numbers and percentages. We used χ^2^ test for categorical variables and z-test for comparison of two proportions. Mann–Whitney U test was used for ordinal variables. *p* value <0.05 was considered as statistically significant. Exponential trend model was used for trend displays.

The study was approved by Ethical Committee of the University of Split (No: 2181-147-01/06/I.D.H.-17-2) and procedures were in accordance with the Helsinki Declaration.

## 3. Results

### 3.1. Acute Intoxications in Children Aged 0–18 Years at Department of Pediatrics, University Hospital of Split over the Period from 2016 to 2021

There were 218 children hospitalized at Department of Pediatrics, University Hospital of Split from 1 January 2016 to 31 December 2021 for acute intoxications, 147 (67.43%) of them for intoxications with various other substances (non-AAIs) and 71 (32.57%) for AAIs.

**Figure 1:**
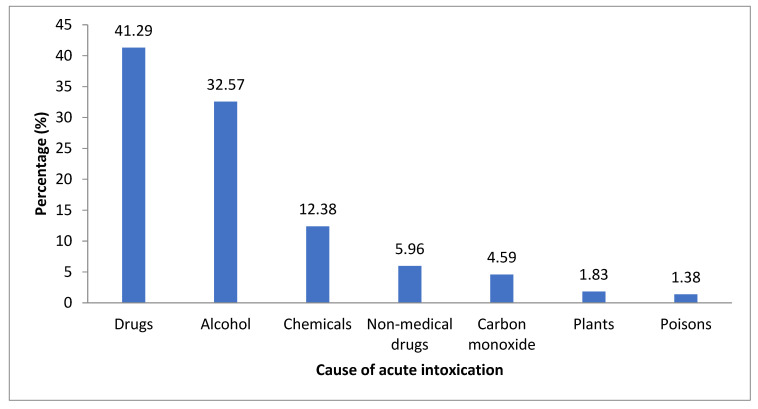
Causes of acute intoxications among children aged 0–18 years hospitalized at the Department of Pediatrics, University Hospital of Split over the period from 1 January 2016 to 31 December 2021 (N = 218).

Drugs (medicaments) were the most common cause of intoxications (41.29%), followed by alcohol (32.57%), chemicals (12.38%), and non-medical drugs, such as marijuana, cocaine, and amphetamine (5.96%), as shown in Figure 1. Poisons (insecticides, rodenticides, and organophosphates) were the rarest cause of intoxications, as shown in Figure 1.

Among children hospitalized for non-AAIs, there were 88 (59.86%) girls and 59 (40.14%) boys. The median age in boys was 3.70 (IQR: 2.20–12.70) years, significantly lower than the median age in girls, which was 14.15 (IQR: 3.70–15.80) years (Mann–Whitney U test; *p* = 0.004).

The age groups with the largest number of children with non-AAIs were the 0–5 (N = 64; 43.54%,) and 14–18 years age groups (N = 60; 40.82%) old, χ^2^ = 69.95, *p* < 0.001. In the youngest age group (0–5 years) there were more boys than girls, while in the oldest age group (14–18 years), there were more girls than boys hospitalized for non-AAIs (χ^2^ = 9.61, *p* = 0.02, Table 1).

In the youngest age group (0–5 years), the most common causes of intoxications were drugs (51.56%) and chemicals (32.81%), similar to intoxications among children in the 6–9 years and 10–13 years age groups, as shown in Table 2. Medicaments were also the most common cause of non-AAIs in the 14–18 years age group (66.67%), followed by non-medical drugs, as shown in Table 2.

Among 90 children with acute drugs poisoning, eight of them were intoxicated with antidepressants, seven with analgesics, seven with antipsychotics, six with antihypertensive drugs, three with antiepileptic drugs, three with antidepressants and antipsychotics, three with more than two different group of drugs, and 11 with other drugs (allopurinol, oxymetazoline, naphtazoline, montelukast, anticholinergics, salbutamol, dimetinden maleate, and iodine). Among 17 children (18.89%), it was intentional intoxication as suicide attempt; 15 of these children were girls and there were only two boys. Marijuana, amphetamines, opioids, cocaine, and hallucinogens were the non-medical drugs thatcaused intoxications among our respondents.

### 3.2. Acute Alcohol Intoxications Among Children Aged 0–18 Years at Department of Pediatrics, University Hospital of Split over the Period from 2016 to 2021

**Figure 2:**
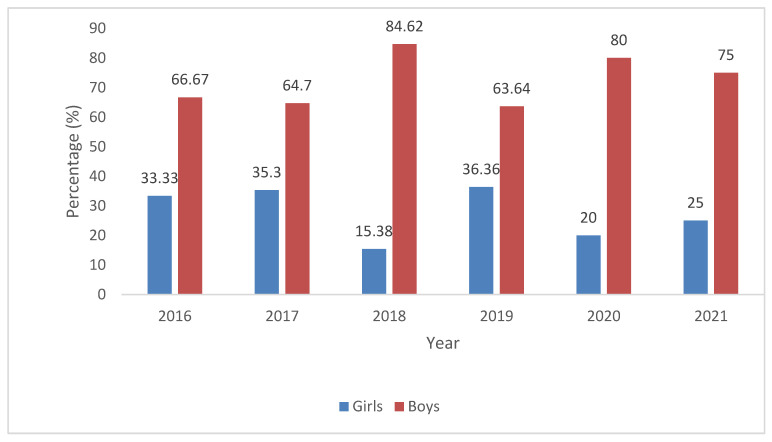
Gender distribution of children aged 0–18 years hospitalized for acute alcohol intoxications at Department of Pediatrics, University Hospital of Split from 1 January 2016 to 31 December 2021 by every observed year.

There were 71 children hospitalized for AAIs in the observed period. Among them, there were 50 (70.42%) boys and 21 (29.58%) girls (χ^2^ = 11.85; *p* < 0.001). Every year, there were more hospitalized boys than girls, as shown in Figure 2. In year 2018, 11 (84.62%) boys and only two (15.38%) girls were hospitalized for AAIs, χ^2^ = 6.23; *p* = 0.013, as shown in Figure 2.

The average age of children hospitalized for AAIs was 16.17 ± 1.30 years; the lowest was 12 and the highest was 17.9 years. The most common age of children (92.96%) was 14–18 years old, and there were no children younger than 9 (χ^2^ = 52.41, *p* < 0.001), as shown in Table 3.

The average BAC was 2.15 ± 0.539‰, without significant difference depending on the gender (*p* = 0.625). The lowest BAC was 0.40‰ and the highest was 3.80‰.

There were six (8.45%) children hospitalized for AAIs who were positive for addictive substances: three for cannabis, two for benzodiazepines, and one for both cannabis and benzodiazepines.

Seventeen (23.94%) children conceived some type of alcohol-related injury.

Twenty (28.17%) children received an examination and/or consultation with a psychologist/psychiatrist during hospitalization for AAIs.

Alcohol consumption was most common on weekends (Fridays to Sundays) and holidays (81.70%) compared to working days (13.80%; χ^2^ = 25.52; *p* < 0.001), and in most cases, it occurred outside children’s homes (72.88%; ≤2 (df = 1) = 12.36; *p* < 0.001). Drinking spirits most often led to intoxications (76.31%), followed by drinking mixed drinks (18.42%), while drinking wine and beer less often resulted in AAIs (5.27%) (χ^2^ (df = 3) = 55.6; *p* < 0.001).

**Figure 3:**
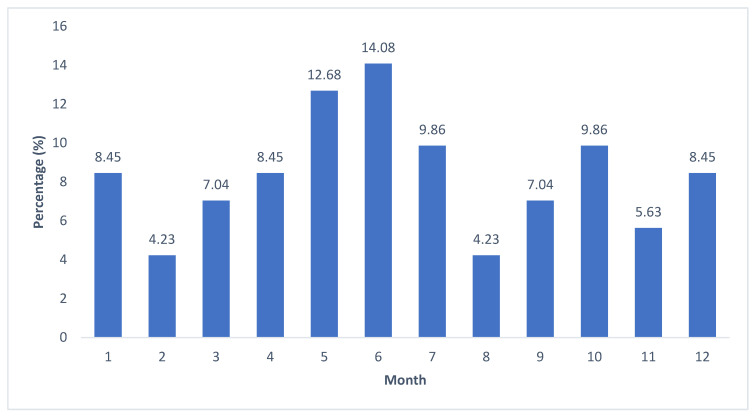
Distribution of hospitalizations for acute alcohol intoxications in children aged 0–18 years at Department of Pediatrics, University Hospital of Split over the period from 1 January 2016 to 31 December 2021, by months.

Children who became intoxicated with alcohol most often consumed it in June (N = 10; 14.08%) and May (N = 9; 12.68%) and less frequently consumed it in February (N = 3; 4.23%) and August (N = 3; 4.23%), as shown in Figure 3.

**Figure 4:**
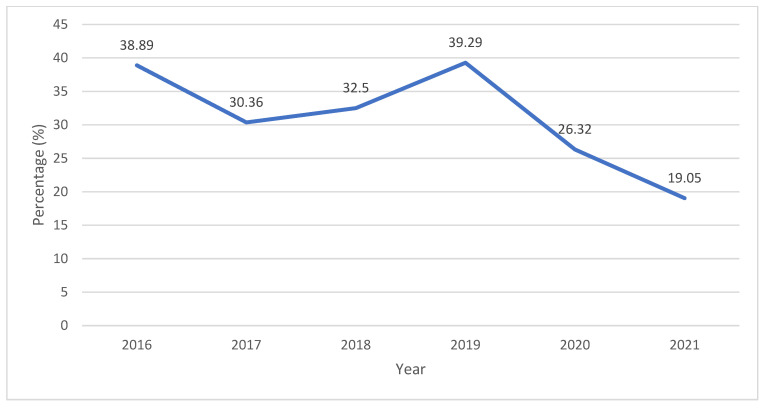
The proportions of hospitalizations for acute alcohol intoxications among all hospitalizations for all types of intoxications in children aged 0–18 years at the Department of Pediatrics, University Hospital of Split, over the period from 1 January 2016 to 31 December 2021.

Generally analyzing, there was no significant decrease in the proportion of hospitalizations due to AAIs among hospitalizations for all types of intoxication in the period from 2016 to 2021 (*p* = 0.104). However, a significant decrease was recorded in 2020 (during the COVID-19 pandemic), when the amount of hospitalizations for AAI was 26.32%, compared to 2019 (the pre-pandemic period), when that amount was 39.29% (z test; *p* = 0.025), as shown in Figure 4. During the pandemic period, the number of children who were hospitalized was also reduced compared to the period before (1729 children in 2021 and 1642 children in 2020 vs. 2376 children in 2019 and 2562 children in 2018).

**Figure 5:**
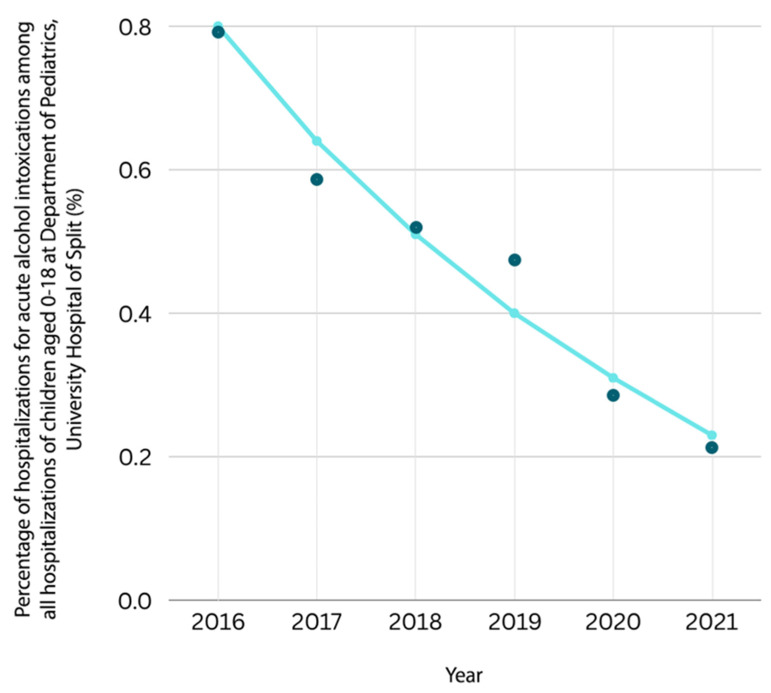
Estimated trend model of proportion of hospitalizations for acute alcohol intoxications among all hospitalizations of children aged 0–18 at Department of Pediatrics, University Hospital of Split from 1 January 2016 to 31 December 2021. OH = 0.81% ∗ 0.79^time^. α = 0.81—expected trend value in year 2016. β1 = 0.79—in every further period, a decline of 21.26% of alcohol intoxications is expected (because (0.7874 − 1.00) ∗ 100 = −21.26%).

In the observed period, a significant decrease in the number of hospitalizations due to AAIs among the total number of all hospitalizations of children aged 0–18 years was recorded, from 0.80% in 2016 to 0.23% in 2021 (Figure 5). According to these results, an annual decline of 21.26% in hospitalizations for AAIs among all types of hospitalizations is expected, as shown in Figure 5.

## 4. Discussion

The results of our study showed a decline in the number of children hospitalized for AAIs among all hospitalizations of children aged 0–18 years at the Department of Pediatrics, University Hospital of Split over the period from 2016 to 2021, especially during COVID-19 pandemic. On the other hand, intoxications with medicaments became a leading cause of acute poisoning in children.

The University Hospital of Split is the clinical hospital center to which all population of Split-Dalmatia County (SDC) and even some surrounding areas gravitate. In year 2021, there were about 423 407 inhabitants in SDC [28]. Bitunjac and Saraga found an alarming increasing trend of hospitalizations for AAIs among children hospitalized at the Department of Pediatrics, University Hospital of Split, from 0.30% in 1997 to 1.70% in 2007 [3]. Another study at the same department showed a declining trend in the following years, from 1.33% in 2008 to 0.75% in 2015, predicting an annual decline of 10.10% in every following year [26]. The results of our study confirmed a continuing declining trend, showing a decrease in hospitalizations for AAIs from 0.80% in 2016 to 0.23% in 2021. Based on these results, a further predicted annual decline in AAIs among all hospitalizations is 21.26% per year, which is even higher than the previous study predicted [26]. The declining trend could be a result of a more pronounced awareness in society about the problems of alcohol consumption among children and adolescents, which influenced the development of better educational or preventive measures in recent years. The living conditions have improved in recent years, and parents and children probably have better knowledge about the negative impact of alcohol consumption on children’s health than before. Drinking habits could also be changed, and it is possible that adolescents still consume alcohol while socializing with their friends, but in smaller quantities that do not lead to AAIs. On the other hand, it could also be possible that some changes in social behaviors among children and adolescents have happened and that drinking alcohol has become less popular than engaging in some other risky behaviors (e.g., e-cigarettes smoking, consuming new drugs, filming risky videos and posting them to social media, etc.).

A significant decline in the number of hospitalizations for AAIs at Department of Pediatrics, University Hospital of Split was reported in the COVID-19 pandemic period. In the year 2020, of all children hospitalized for acute intoxications, 26.32% were hospitalized for AAIs, while a year before, 39.29% were hospitalized for AAIs (*p* = 0.025). While some other studies found an increase in AAIs among children older than 15 years in the first months of the pandemic [29], others also reported a decline in AAIs during the pandemic period [30,31,32,33,34]. A decline in AAIs at the Department of Pediatrics, University Hospital of Split during the COVID-19 pandemic is probably the result of reduced alcohol consumption among adolescents during the lockdown period, as reported in a study provided among high school students in SDC [32]. That study found that 44.76% of adolescents in SDC consumed alcohol during the lockdown period, while 84.66% of them were drinking before the lockdown period [32]. The frequency of drinking also decreased significantly [32,33]. During the COVID-19 pandemic period, adolescents were hanging out with their friends less often, and drinking with friends was also significantly reduced compared to a period before [32]. Reduced peer pressure and the importance of peer relationships due to school closure during the lockdown period could also result in the decline in drinking among children [33]. The availability of alcoholic beverages was limited because numerous stores were closed. Also, a general decrease in number of hospitalizations for various reasons during the pandemic period was reported, as in some other studies [35,36]. It is possible that fewer children were hospitalized for diagnostic procedures during the pandemic period than before pandemic because of epidemiological situation and fear of spreading COVID-19 infection. The decrease in the number of hospitalizations and examinations in emergency departments could also be the result of the fear of becoming infected with COVID-19 in the case of seeking medical attention. In accordance with that, some studies found a significant increase in AAIs after the lockdown period [37], when strict prohibition measures ended and the fear of infection was lower. On the contrary, a further decline in AAIs among children and adolescents in the following years was reported in our study (from 0.30% in 2020 to 0.23% in 2021), confirming the long-term general declining trend in hospitalizations for AAIs among children and adolescents in Split-Dalmatia County during recent years.

AAIs were most common among children aged 14–18 years, similar to the results of the previous studies at the same department [3,26] and those of other studies [38,39]. The average age was 16.17 ± 1.30 years, which is higher than some other studies reported [24,40]. The average age in the previous studies at the same department was also much younger, 14.01 years over the period from 1997 to 2007 [3] and 15.95 ± 1.51 years from 2008 to 2015 [26].

More boys were hospitalized for AAIs at the Department of Pediatrics, University Hospital of Split than girls in the observed period (70.42% boys vs. 29.58% girls). Over the period from 2008 to 2015, 63.60% of children hospitalized for AAIs were boys [26]. Between the years 1997 and 2007, there were also more boys (71.10%) hospitalized for AAIs than girls, but an increasing trend of hospitalizations among girls was detected [3]. Some studies reported an increase in hospitalizations for AAIs among girls [19,41], while others reported the same frequency among boys and girls [24,42]. In every year of the observed period in our study (from 2016 to 2021), boys were more often hospitalized for AAIs than girls, confirming that boys in Split-Dalmatia County become more intoxicated with alcohol than girls. Boys possibly consume greater quantities of alcohol than girls and, therefore, more often become intoxicated, but it should be further investigated. Maybe they more often drink spirits than girls, resulting in a higher frequency of AAIs among them [27].

Alcohol intoxications among children hospitalized for AAIs at the Department of Pediatrics, University Hospital of Split happened outside their homes in 72.88% cases. Compared to the results of the previous study, where 92.42% intoxications happened outside their homes [26], these results could indicate that in the examined period, more children consumed alcohol at home, maybe due to the restrictive pandemic measures.

The alcohol intoxications were most frequent on weekends and holidays (81.70%), when children and adolescents have more leisure time and socialize with their friends. Similar results were reported in previous studies at the same department [3,26] and studies in other countries [18,43]. Most hospitalizations for AAIs among children in our study occurred in June (14.08%) and May (12.68%). The school year in Croatia usually ends in June or May (for final year high school students). Therefore, the highest rate of intoxications among children in these months could be the result of a higher frequency of socializing with friends at the end of the school year or celebrations of the school year ending. These results indicate some changing trends, because over the period from 2008 to 2015, intoxications were most often seen in August (13.60%) and January (11.76%) [26], when children are on their school break.

A large number of children hospitalized for AAIs at our department had some kind of alcohol-related injuries (23.94%). These results indicate an increasing trend of alcohol-related injuries among children who hospitalized for AAIs at the University Hospital of Split compared to results of the previous studies at the same department [3,26]. Over the period from 2008 to 2015, 11.03% of children who were hospitalized for AAIs had alcohol-related injuries [26], while over the period from 1997 to 2007, 1.67% of children had that kind of injury [3]. This could be due to a higher BAC among children in our study (2.15 ± 0.539‰) than those in a previous study whose BAC was 2.003 ± 0.585‰ [26].

We also found an increasing trend of children hospitalized for AAIs who were positive for drugs at the University Hospital of Split. The results of our study found that 8.45% of children were positive to drugs, while in a study from 2008 to 2015, 6.25% were positive for drugs [26], and from 1997 to 2007, 2.90% of children were positive to drugs [3]. Marijuana was the most common drug, a finding similar to that in some other studies [44]. On the other hand, we found a decline in psychological support though examinations or interventions by a psychiatrist or psychologist of children hospitalized for AAIs. In our study, 28.17% of children received some form of psychological support during hospitalization, far fewer than over the period from 2008 to 2015, when 51.29% of children received that type of support [26]. According to an increasing trend of drug usage among adolescents who become intoxicated with alcohol, psychological support during hospitalizations for AAIs should be included more often. The declining trend of psychological support at our department is not very clear, because the hospital protocols have not changed over time. It may result from the fact that these hospitalizations most often occur on holidays and weekends when psychologists are not available. Also, these children usually go home the day after they are hospitalized, most often before they receive planned psychological support, and some parents refuse psychological support for their children. Maybe some parents and children fear that including them in a psychological evaluation will stigmatize them as psychiatric patients and, therefore, refuse it. We have to keep in mind that some of these children received psychological support after their hospitalization, but the exact data should be further explored. We strongly recommend including psychological support and outpatient psychological monitoring to all children who were hospitalized for AAIs or intentional non-AAIs in order to improve their mental health and prevent further engaging in risky behaviors.

Over the period from 1997 to 2007, the most common cause of intoxications at the Department of Pediatrics, University Hospital of Split was alcohol (40.20%), followed by drugs (36.90%) [3]. A study at the same department over the period from 2008 to 2015 also found alcohol as most common cause of intoxications (55.74%), followed by intoxications with drugs (28.89%) [26]. The results of our study found a significant difference because drugs (medicaments) became the leading cause of intoxications (41.29%), while alcohol was in second place (32.57%). A significant increase in intoxications with non-medical drugs (e.g., marijuana, amphetamine, cocaine, etc.), from 0.30% over the period from 1997 to 2007 [3] and 2.25% from 2008 to 2015 [26] to 5.96% over the period from 2016 to 2021, was also detected. Therefore, the results of our study showed that the most common causes of intoxications in children hospitalized at the Department of Pediatrics, University Hospital of Split are significantly changing over time. Some other new studies also found drugs as the most common cause of intoxications in children [6,45,46], but they reported a significantly lower amount of AAIs among children (1.50% [6] and 4.10% [45]). Matalova et al. reported drug intoxications most often among children younger than 3 years [47]. In this age group, intoxications are most frequently accidental [48]. In our study, non-AAIs were most often observed among boys (56.25%) younger than 5 years and girls (76.67%) aged 14–18 years. Drugs and chemicals were the most common cause of acute poisoning among the youngest children, while drugs and non-medical drugs (marijuana, amphetamines, etc.) were the leading causes among the oldest children. Antidepressants and antipsychotic drugs were the most common drugs, a finding similar to that of some other studies [46]. Among children with drug intoxications, there were 18.89% suicide attempts, mostly among girls. Some studies reported 4.86% of intoxications as suicide attempts [49], also more often in girls [46,49]. Based on the fact that suicide is the second most common cause of death among adolescents [50], these results are alarming. Psychological assessment and support to children in schools and their homes is, therefore, extremely important in the aim to improve their mental health. Communication and early recognition of mental health problems is crucial for the timely initiation of appropriate therapeutic measures. Post-COVID-19 time is particularly challenging, because the lockdown period could have a significant long-term impact on adolescents’ mental health. Some studies reported a higher frequency of anxiety and suicidal thoughts among adolescents during the lockdown period [51,52], and the long-term consequences of social exclusion during the lockdown period are still unknown. Therefore, advancement in improving and monitoring the mental health of children and adolescents by their parents, teachers, and physicians is highly advisable.

### 4.1. Limitations of the Study

This is a retrospective study, and some medical histories lacked some data (e.g., place of intoxication, type of beverages, etc.), so we analyzed only the data we had. In this study, we did not examine the reasons for intoxications and the severity of children’s clinical status. Also, some follow-up of these patients, especially those who received psychological support, could be very important. In addition, we have to keep in mind that probably not all children with intoxications were hospitalized. Some of them could have been examined at local emergency departments, and some of them possibly did not even seek medical help.

### 4.2. Strengths of the Study

This study is a continuation of previous studies at the Department of Pediatrics, University Hospital of Split [3,26]. With the results of these studies [3,26], and comparing them with the results of our study at the same Department, we made a 24-year-long retrospective examination of changes of intoxications among children in a large area of SDC.

Significant changing trends in causes of acute intoxications among children and adolescents in SDC during recent years were found in our study. The prevalence of AAIs among children is showing a decreasing trend, while medicaments became the leading cause of acute intoxications. On the other hand, intoxications with non-medical drugs among children are increasing. These results suggest some new trends in children and adolescents’ behavior patterns and give us insight into their mental health, indicating the need for new preventive and psychotherapeutic measures.

We detected age and gender groups with risk for AAIs and non-AAIs, as well as circumstances for AAIs among children, so the results of this study could help in creating preventive measures for children and adolescents. We also detected a great number of children with medicaments poisonings as suicidal attempts, indicating a need for improving mental wellbeing among adolescents and children. Therefore, providing adolescents with psychological support from their parents, teachers, society, or professionals is also indispensable for their mental wellbeing and the prevention of substance use. Early recognition of children and adolescents with problems, such as alcohol consumption or mental health problems, and early initiation in psychotherapeutic or clinical treatment is also very important for long-term consequences. Limiting the availability of alcohol to children [53] and prohibiting advertising alcoholic beverages on social media [54], as well as implementing better educational programs in schools [55] to teach children about the negative consequences of substance use, could prevent them engaging in risky behaviors.

## 5. Conclusions

The main causes of intoxications in children are changing over time. Alcohol intoxications, luckily, seem to have a decreasing trend, but the increasing trend regarding drug (medicaments) and non-medical drug intoxications, especially those taken as suicidal attempts, should warn us that better preventive measures and investments in the improvement of the mental health of children and adolescents, especially after the COVID-19 pandemic, are crucial. The development of preventive educational programs in schools and society, limitations on the availability of alcohol and other substances to children, strong psychosocial support to children and adolescents, and the early initiation of clinical and psychotherapeutic assessment could prevent children engaging in risky behaviors. Investment in improving mental health among children and adolescents, early recognition (by parents, teachers, and physicians) of mental health problems, early psychotherapeutic treatment of children with mental health problems, and restriction of access to substances that could cause acute intoxication to children could lead to a decrease in acute intoxications among children and adolescents.

## Figures and Tables

**Table 1:** Age and gender distribution of children hospitalized for non-alcohol intoxications at Department of Pediatrics, University Hospital of Split over the period from 1 January 2016 to 31 December 2021 (N = 147).

Age Group (Years)	Boys N (%)	Girls N (%)	Total N (%)
0–5	36 (56.25)	28 (43.75)	64 (100)
6–9	4 (44.44)	5 (55.56)	9 (100)
10–13	5 (35.71)	9 (64.29)	14 (100)
14–18	14 (23.33)	46 (76.67)	60 (100)

**Table 2:** Causes of non-AAIs among children aged 0–18 years hospitalized at Department of Pediatrics, University Hospital of Split over the period from 1 January 2016 to 31 December 2021 (N = 147), by age groups.

	Age Group			
Cause of Intoxication	0–5 Years N (%)	6–9 Years N (%)	10–13 Years N (%)	14–18 Years N (%)
Drugs (medicaments)	33 (51.56)	5 (55.56)	12 (85.71)	40 (66.67)
Chemicals	21 (32.81)	3 (33.33)	2 (14.29)	2 (3.33)
Non-medical drugs	1 (1.56)	0 (0.00)	0 (0.00)	12 (20.00)
Carbon monoxide	3 (4.69)	1 (11.11)	0(0.00)	6 (10.00)
Plants	4 (6.25)	0 (0.00)	0 (0.00)	0 (0.00)
Poisons	2 (3.13)	0 (0.00)	0 (0.00)	0 (0.00)
Total	64 (100)	9 (100)	14 (100)	60 (100)

**Table 3:** Age and gender distribution of children hospitalized for acute alcohol intoxication at Department of Pediatrics, University Hospital of Split over the period from 1 January 2016 to 31 December 2021, according to gender and age (N = 71).

Age Group (Years)	Boys N (%)	Girls N (%)	Total
0–5	0 (0.00)	0 (0.00)	0 (0.00)
6–9	(0.00)	0 (0.00)	0 (0.00)
10–13	3 (60.00)	2 (40.00)	5 (100)
14–18	47 (71.21)	19 (28.79)	66 (100)

## Data Availability

The datasets analyzed during the current study are available from the corresponding author on reasonable request.

## Abbreviations

AAIs	acute alcohol intoxications
BAC	blood alcohol concentration
non-AAIs	non-alcohol intoxications
SDC	Split-Dalmatia County
